# Identification of aspirin and diclofenac binding proteins in the red complex pathogens

**DOI:** 10.6026/97320630017192

**Published:** 2021-01-31

**Authors:** Geethika Babu, Veeraraghavan Vishnu Priya, Pothapur Keshaav Krishnaa, Rengasamy Gayathri, Jayaseelan Vijayashree Priyadharsini

**Affiliations:** 1 Department of Biochemistry, Saveetha Dental College, Saveetha Institute of Medical and Technical Sciences (SIMATS), Saveetha University, Chennai 600077, India; 2 Biomedical Research Unit and Laboratory Animal Centre-Dental Research Cell, Saveetha Dental College, Saveetha Institute of Medical and Technical Sciences (SIMATS), Saveetha University, Chennai 600077, India

**Keywords:** aspirin, diclofenac, proteins, pathogens

## Abstract

Red complex organisms are a group of organisms (Porphyromonas gingivalis ATCC 33277, Treponema denticola ATCC 35405, Tannerella forsythia ATCC 43037) that have been identified for the causation of periodontal diseases. Aspirin and diclofenac have been used as regular analgesics. Therefore, it is of interest to document the identification of aspirin and diclofenac binding proteins in the red complex pathogens using the STITCH v.5 pipeline. The virulence properties of these proteins were analyzed using VICMPred and VirulentPred software. Thus, we document 000 number of proteins having optimal binding features with the known analgesics.

## Background

Periodontal infection is one of the most common dental infections that occur [1] second to dental caries. The first and initial process that is involved in the process of periodontitis is the colonisation of oral pathogens to form dental plaque [2]. There are various factors that are involved in the formation of biofilm such as the acidity of the oral environment and virulence of the bacteria and factors such as host immune response to inflammation plays a central role in disease pathogenesis [3]. Periodontitis can be defined as an inflammatory disease of supporting tissues of teeth caused by specific microorganisms or groups of specific microorganisms, resulting in progressive destruction of the periodontal ligament and alveolar bone with periodontal pocket formation, gingival recession or both [4]. Recent advances have suggested that the varied microbial environmental present within the biofilms promotes accelerated genotypic and phenotypic diversity that provides a form of cover that can safeguard the microbial colonies in the face of adverse conditions, such as those faced by pathogens in the host [2]. Periodontal diseases are basically bacterial infections associated with a complex microbial flora associated with that o of the dental biofilm. This flora is composed predominantly of strictly anaerobic Gram-negative species that will in-turn produce a local and a systemic inflammatory response, ultimately leading to periodontal tissue destruction [5-7]. However, only few species such as Aggregatibacter actinomycetemcomitans (A.a) and Porphyromonas gingivalis have been classically grouped as periodontal pathogens [8,9] Socransky et al. showed that periodontal diseases such as gingivitis and periodontitis are associated with a group of microbes rather than individual pathogens present at the periodontal sites. It comprises a consortium of three species namely, Tannerella forsythia, P. gingivalis and Treponema denticola which has been considered the most pathogenic microbial complex [5,10]. There have been various modalities of treatment of periodontitis, one of the treatment modalities is the use of therapeutic agents such as analgesics to obtain symptomatic relief, a few commonly used include aspirin and diclofenac for periodontitis [11,12].

## Materials and Methods:

### Study design:

**Figure 1:**
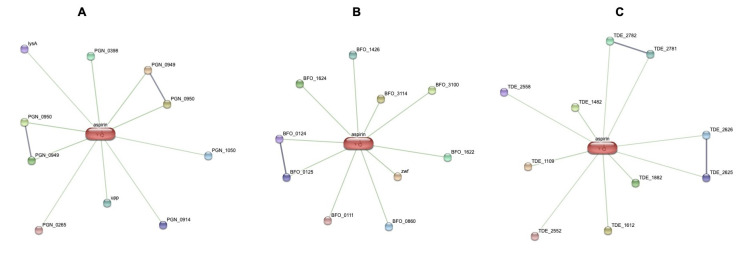
Interaction network of Aspirin with protein repertoire of red complex pathogens (A) Porphyromonas gingivalis (B) Tannerella forsythia and (C) Treponema denticola.

The reaction as well as interaction of the compound with protein of bacteria was analyzed using STITCH v.5 pipeline13 (Figure 1) and therefore the virulence properties of these interacting proteins were deduced and analysed by VICMPred14 and VirulentPred softwares [15]. Porphyromonas gingivalis ATCC 33277, Treponema denticola ATCC 35405, Tannerella forsythia ATCC 43037 were the strains of red complex pathogens that were utilized in this study. These strains were included within the STITCH database, and therefore the query was user defined.

### Prediction of protein-drug interactions:

STITCH database (Version 5; 2016) is an extensive platform for various predicted or known interactions between provides a comprehensive platform for known and predicted interactions between various compounds and proteins. The interactions between the compounds and the organism could vary from direct or physical and indirect or functional associations, which primarily arise from computational prediction and from interactions aggregated from various other (primary) databases (Figure 1). The repertoire of proteins, which interact with P. gingivalis, T. denticola, and T. forsythia, were further utilised for predicting virulence [13].

### Virulence prediction:

**Figure 2:**
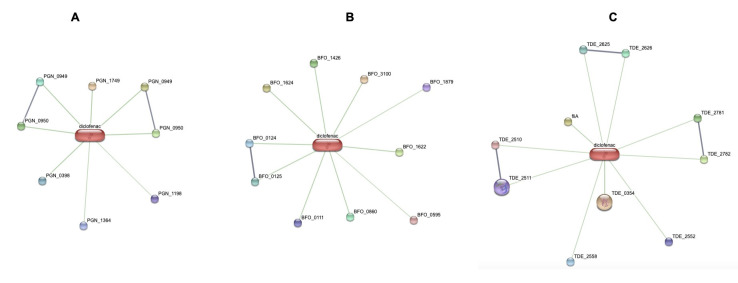
Interaction network of Diclofenac with protein repertoire of red complex pathogens (A) Porphyromonas gingivalis (B) Tannerella forsythia and (C) Treponema denticola.

VICMpred [14] and VirulentPred [15] pipelines were used for the identification of virulence factors targeted by aspirin and diclofenac among red complex pathogens. These tools employed support vector machine (SVM)-based five-fold cross-validation process to validate results. Virulence factors were screened on the idea of proteins using VirulentPred tool, which classified them into two groups' namely virulent and avirulent factors. VICMpred categorises proteins into four major classes, such as, proteins involved in cellular process, metabolism, information storage, and virulence. The general potent accuracies of VICMpred and VirulentPred servers were 70.75% and 86%, respectively. The FASTA formats of the actual proteins were retrieved from the NCBI database and were used as an input to run the algorithm (Figure 2) [15,16].

### Prediction of subcellular localization of the virulent proteins:

The prediction of localisation of proteins at a sub cellular level helps in designing unique drug targets or for substantiating the role of an antimicrobial agent, which targets the virulent protein. Cell surface proteins are considered to be of great interest, as they will be used as vaccine targets. PSORTb V3.0 is an algorithm, which assigns a probable local site to a protein from an aminoalkanoic acid sequence that's provided [17].

### Prediction of B-cell epitopes within the virulent proteins:

**Figure 3:**
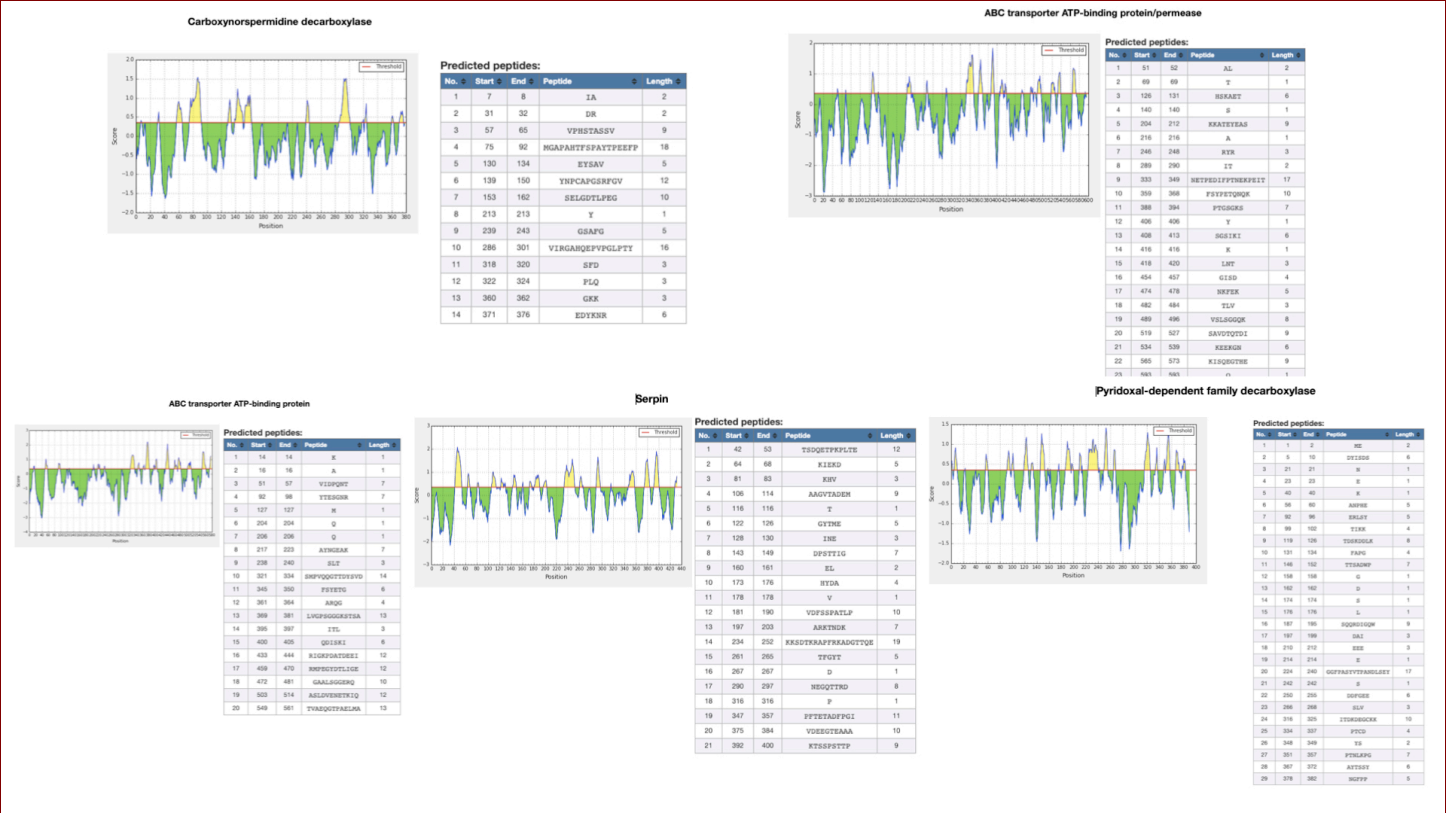
Epitopes identified in the virulent proteins of red complex pathogens interacting with Aspirin.

The BepiPred-2.0 server predicts B-cell epitopes from a protein sequence, employing a Random Forest algorithm on the idea of epitopes and non-epitope amino acids determined from crystal structures. The residues with scores above the edge (>0.5) are predicted to be a part of an epitope and colored in yellow on the graph (Figure 3)[18,19].

## Results and Discussion:

The STITCH pipeline was used to identify the protein interaction between red complex bacteria and the compounds, Aspirin and Diclofenac (Figure 1). Further each of the protein that was found to be interacting with the compounds was assessed for their virulence property using VirulentPred and VICMpred. The scores produced by the algorithms confirmed the nature of the proteins and grouped them into two classes, virulent and avirulent. Proteins interacting with aspirin were primarily related to metabolism processes, followed by cellular process. There were no proteins related to information storage or virulence factor that were identified that interacted with aspirin. The scores from VirulentPred marked carboxynorspermidine decarboxylase as virulent factors (Figure 1; Tables 1 and 2 - see PDF). Out of the proteins reacting with Diclofenac majority of them were related to metabolism followed by cellular process and virulence factor. The scores from VirulenPred implied peptidyl-prolyl cis-trans isomerase cyclophilin type as the virulent Protein (Figure 1; Tables 1 and 2 - see PDF). STITCH prediction for Aspirin revealed proteins mainly associated with metabolism and cellular processes. None for virulence factor and information storage were retrieved .Two compounds such as Pyridoxyl dependent family decarboxylase and ABC transporter ATP- binding protein/permease, associated with metabolism were found to be virulent based on the score obtained from VirulentPred (Figure 2; Tables 1 and 2 - see PDF). When looked at interaction with Diclofenac most of the proteins that were retrieved belonged cellular process. Based on the scores obtained from VirulentPred ABC transporter ATP binding protein/ permease, related to metabolism was found to be virulent (Figure 2; Tables 1 and 2 - see PDF). Proteins interacting with aspirin, majority belonged to metabolism, followed by cellular process and virulence factor as retrieved from STITCH prediction. A protein, serpin associated with metabolism and a protein ABC transporter ATP- binding protein, associated with metabolism were predicted to be associated with virulence as per the scores obtained from VirulentPred. On interaction with Diclofenac, most of the interacting proteins belonged to metabolism. As per the scores obtained from VirulentPred, no protein was found to be virulent with respect to Diclofenac. Aspirin has been used through the ages in periodontitis patient and has been attributed to its anti inflammatory property [11,[Bibr R12]]. Aspirin has been used as an adjunct to standard periodontal therapy and has yielded good improvements in periodontal health [20,21]. This is a first of its kind study, as the antibacterial property of Aspirin has never been reported in litreature against periodontal pathogens. Diclofenac has also been used in the post periodontal treatment and is highly debated for its usage attributed to its analgesic property [22,23]. The use of Diclofenac as an antibacterial agent has been reported against bacteria such as Salmonella typhimurium [24], Escherichia coli [25], and Listeria and Mycobacterium tuberculosis. [26] However the same hasn't been tested against red complex pathogens. Thus this study is a first of its kind in the field. Faizuddin et al. [27] has reported improvement in periodontal health and lesser periodontal attachment loss in patients with long-term aspirin usage. The improvement of periodontal health is attributed to reduced bleeding on probing which could have been due to the antiplatelet activity. The lower level of attachment loss is not accounted for and could be due to the modification of the bacterial flora with the intake of aspirin, but the same has to be evaluated clinically. Kim et al. [28] reported reduction in the pocket depth on usage of aspirin for about a week and attributed the same to a reduction in inflammation attributed to the aspirin, the antibacterial property could also play a role in the same. In a study conducted by Fraser et al.[29] it has been reported that when antibiotic and aspirin as used against minor respiratory infections in two separate groups they yield similar results. This is proof to the fact that Aspirin has antibacterial property. Wang et al. [30] has reported anti bacterial property of aspirin against H.pylori and has reported that this could be due to the acidic nature of aspirin but that could not solely be the reason. They also reported that aspirin inhibits the growth of H.pylori. When in the diclofenac in considered, Milani et al. [31] have reported antibacterial property of diclofenac against E.faecalis which is one of the most common organisms involved in root canal treatment failure. The reason for the antimicrobial property of Diclofenac could be due to inhibition of bacterial DNA synthesis [32] or due to impairment of membrane activity [33,34] but the underlying mechanism is still unclear. In the present study these compounds have been screened for potential targets in red complex pathogens namely P.gingivalis, T.denticola and T.forsythia. Aspirin is found to be active against all three organisms whereas diclofenac is found to be inactive against T.forsythia. The protein ABC transporter ATP binding protein is found virulent in the case of aspirin and diclofenac with T.denticola and aspirin against T.forsythia. Matsson et al. [35] have reported novel drug delivery regarding this family of transporters, hence further studies can be carried to see if the same relation holds good in the case of periodontal pathogens. Vijayashree, 2019, has recently used the virtual screening methods to identify potential targets of non-steroidal anti-inflammatory drugs acetaminophen and ibuprofen against red complex pathogens [36]. Also, the authors analyzed certain phytocompounds using computational approach to identify targets in dental pathogens [37]. Hence, such in silico studies can reduce the time and cost required to assess the antimicrobial effect of bioactive compounds. The compounds validated using this procedure can be further screened using experimental procedures and clinical studies using animal and human volunteers. The present study is a unique who shows quintessential protein interactions of two commonly used drugs, aspirin and diclofenac against red complex pathogens. There are a few limitation of the study such that the reactions may be purely physical and may not hold any functional significance, the same protein interaction may not hold good in an invivo environment, certain host proteins may mimic these proteins and thus should be taken care while using targeted drug therapy.

## Conclusion

We document the identification of aspirin and diclofenac binding proteins in the red complex pathogens using the STITCH v.5 pipeline for further consideration.

## Figures and Tables

**Figure 4:**
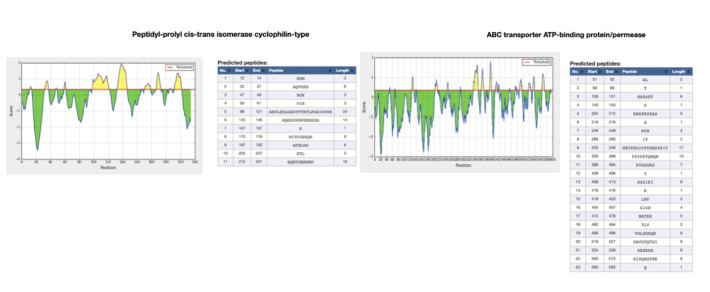
Epitopes identified in the virulent proteins of red complex pathogens interacting with diclofenac.
